# The role of digital literacy in achieving health equity in the third millennium society: A literature review

**DOI:** 10.3389/fpubh.2023.1109323

**Published:** 2023-02-20

**Authors:** Laura Leondina Campanozzi, Filippo Gibelli, Paolo Bailo, Giulio Nittari, Ascanio Sirignano, Giovanna Ricci

**Affiliations:** ^1^Research Unit of Bioethics and Humanities, Campus Bio-Medico University of Rome, Rome, Italy; ^2^Section of Legal Medicine, School of Law, University of Camerino, Camerino, Italy; ^3^Telemedicine and Telepharmacy Centre, School of Medicinal and Health Products Sciences, University of Camerino, Camerino, Italy

**Keywords:** digital literacy, computer literacy, digital health, digital divide, e-health literacy

## Abstract

Healthcare in the third millennium is largely delivered through systems involving the use of the technological devices and services, foremost among them telemedicine. For the adequate delivery of digital medicine services, however, it is necessary for users to be digitally literate, that is, able to consciously make use of technology. In order to understand how relevant digital literacy is in determining the effectiveness of e-Health services, we performed a traditional literature review on 3 major databases by combining the terms “Digital Literacy” and “Computer Literacy” with the terms “Telemedicine” and “Telehealth”. Starting from an initial library of 1,077 papers, we selected 38 articles. At the outcome of the search, we found that digital literacy is a pivotal element in conditioning the effectiveness of telemedicine and digital medicine services in general, however, with some limitations.

## 1. Introduction

The phrase “digital literacy” was coined in 1997 by Paul Gilster, who, in his book entitled “Digital Literacy” defined it as “*the ability to understand and use information in multiple formats from a wide variety of sources when it is presented via computers”* (1). According to Wilhelm, a digitally literate person should be able to “*access, manage, integrate, evaluate, and create information”* (2). A further contribution to the clarification of the concept of digital literacy came in 2009, when Cornell University proposed a new definition: “*the ability to find, evaluate, utilize, share, and create content using information technologies and the Internet”* (3). In 2013, the American Library Association defined it as “*the ability to use information and communication technologies to find, evaluate, create, and communicate information, requiring both cognitive and technical skills”* (4). Being digitally literate, in essence, means possessing the skills necessary to be able to live within a society in which communication is increasingly based on new technologies.

When thinking about the topic of digital literacy, it comes naturally in the first instance to refer to the more purely social aspects of community life, such as relating to others, communicating, and interfacing with others through digital tools not accessible to those who have not mastered their use. In a society where technology increasingly permeates all aspects of life, such as that of the third millennium, however, digital literacy also means access to several healthcare services. Indeed, the advancement of technology in recent decades has led to the emergence of the concept of “digital health”, defined by the WHO as “*a broad umbrella term encompassing eHealth (which includes mHealth), as well as emerging areas, such as the use of advanced computing sciences in “big data”, genomics and artificial intelligence”* (5).

In 2006, Norman and Skinner (6) proposed a conceptual model encompassing six different literacy domains needed to process information from technological sources: traditional literacy, health literacy, information literacy, scientific literacy, media literacy and computer literacy. According to the authors' view, health literacy consists of the ability to perform basic reading and numeracy tasks necessary to function in the health care environment, so individuals with adequate health literacy are able to read, understand and act on health information. In fact, the term “health literacy” had been already defined in 2000 as “*the degree to which individuals can obtain, process, understand, and communicate about health-related information needed to make informed health decisions”* (7). Here, then, is where digital literacy applied to medicine represents what can be called “e-Health literacy” (electronic health literacy), which can be measured and quantified through scales, such as that devised by Norman and Skinner themselves (eHEALS: e-HEAlth Literacy Scale), the best known and most widely used (8). E-Health literacy is one of the key tools to counter the so-called “digital divide”, which today translates not simply into the inability of some citizens to access information, but into the preclusion of access to actual health services. It is therefore necessary to understand how today, in the third millennium, digital literacy is much more than a simple technological know-how, but represents a real tool at the service of citizenship, enabling individuals to have equal access to numerous categories of services, including health services.

Precisely because of its growing impact in terms of access to health services, in recent years digital literacy has been universally recognized as falling squarely within the SDOH (Social Determinants of Health), that are non-strictly medical elements that influence a wide range of health outcomes and risks, as well as functioning and quality of life. A major driving force behind the amplification of the primary role played by technology in the delivery of health care services was undoubtedly the COVID-19 pandemic, which by imposing a restriction on interhuman contact forced the adoption of health care measures that could be delivered remotely, by means of technological tools (9). And indeed, it is sufficient to think about the SARS-CoV-2 pandemic to realize how preeminent the role of e-Health literacy is in modern healthcare. Booking an anti-COVID vaccine or swab, accessing the service's website from which to download the test result, booking a blood test to determine the level of antiviral antibodies, accessing the Ministry of Health's website to download the vaccine certificate, interacting *via* e-mail with the primary care physician: these are all tasks that many of us take for granted, but they require a certain level of digital literacy to accomplish properly.

It is abundantly clear that the problem predominantly affects certain categories of people (the elderly, people with a low cultural level, people living in rural areas, where the access to technology is limited), but one of the cornerstones of an evolved society is equity in healthcare provision, and today, in the third millennium, it seems really difficult to think that this goal can be pursued apart from a serious and structured digital literacy policy.

Since health is a basic human right, the delivery of health services must be done equitably, that is, by ensuring that all individuals could reach their full potential for health and wellbeing. The digital divide brought about by the increasingly pervasive diffusion of technology in health care necessitates the implementation of increasingly efficient education programs in the use of new digital tools, as it has become clear that education is a primary tool for ensuring health equity, no less important than scientific progress (10, 11).

## 2. Aims and objectives

Implementing the quality of the technologies underlying telemedicine services and e-Health services in general without promoting at the same pace the knowledge of the use of IT and digital tools is clearly a strategy destined to fail. According to Watts' claim, in fact, “*any healthcare development that doesn't rapidly become available to all individuals has the unintended consequence of fuelling health inequality”* (12).

This paper aims to define, through a review of the literature of the past 10 years, the extent of the impact of digital literacy on access to telemedicine services. In other words, we sought to understand whether and to what extent high or low levels of digital literacy are involved in determining the extent to which telemedicine services are used.

## 3. Materials and methods

### 3.1. Searching strategy

The research was carried out on the scientific literature between January 2011 and October 2022 in the online databases of PubMed, Scopus, and Web of Science (WoS). The search was performed entering the following base string: (“Digital Literacy” OR “Computer Literacy”) AND (“Telemedicine” OR “Telehealth”), limiting the results to English-language articles published in the above time frame. The basic string was, of course, modified according to the individual peculiarities of the various databases on which the search was carried out. We voluntarily omitted to include the databases for the gray literature search to avoid contaminating the review with papers with uncertified scientific validity.

We performed a preliminary skimming independently. Each author read the abstracts of the articles and identified those they considered useful for the review. At the end of the preliminary evaluation phase, the authors discussed the selected articles, debating the suitability of the individual papers. At the end of the selection procedure, the authors read all the articles in order to collect the data for the review.

### 3.2. Selection criteria

The research initially provided 1,077 results. Specifically, 223 papers were found in PubMed, 682 in Scopus, and 172 in Web of Science. The types of study objects of interest were original researches, review articles, and perspective papers. We made an initial pre-selection by removing duplicate papers (*n* = 453) and articles for which the full text was not available (*n* = 7). We then read the titles and abstracts of the remaining 617 articles in order to identify papers suitable for reading the full text. A total of 94 articles were discarded just by reading the titles, which were clearly not in line with the purpose of the review. After reading the abstracts of the remaining 523 papers, we excluded an additional 293 papers: 107 because they illustrated particular telemedicine services without a clear discussion of the link to digital literacy, 112 because they delved into the topic of digital literacy without particular reference to its impact on access of telemedicine services, and an additional 74 because, although they dealt with the impact of digital literacy on access to telemedicine, they did not provide useful insights to answer the review questions.

With regard to the 112 papers excluded because they lack clear references to telemedicine, we would like to point out how we nevertheless decided not to exclude some of the papers focused almost exclusively on digital literacy without direct reference to telemedicine because they related to older age groups, thus of particular interest in answering the review questions.

We then proceeded to read the full text of the remaining 230 papers, of which we decided to include in the review the 37 that we considered most valid and scientifically accurate.

**Figure 1:**
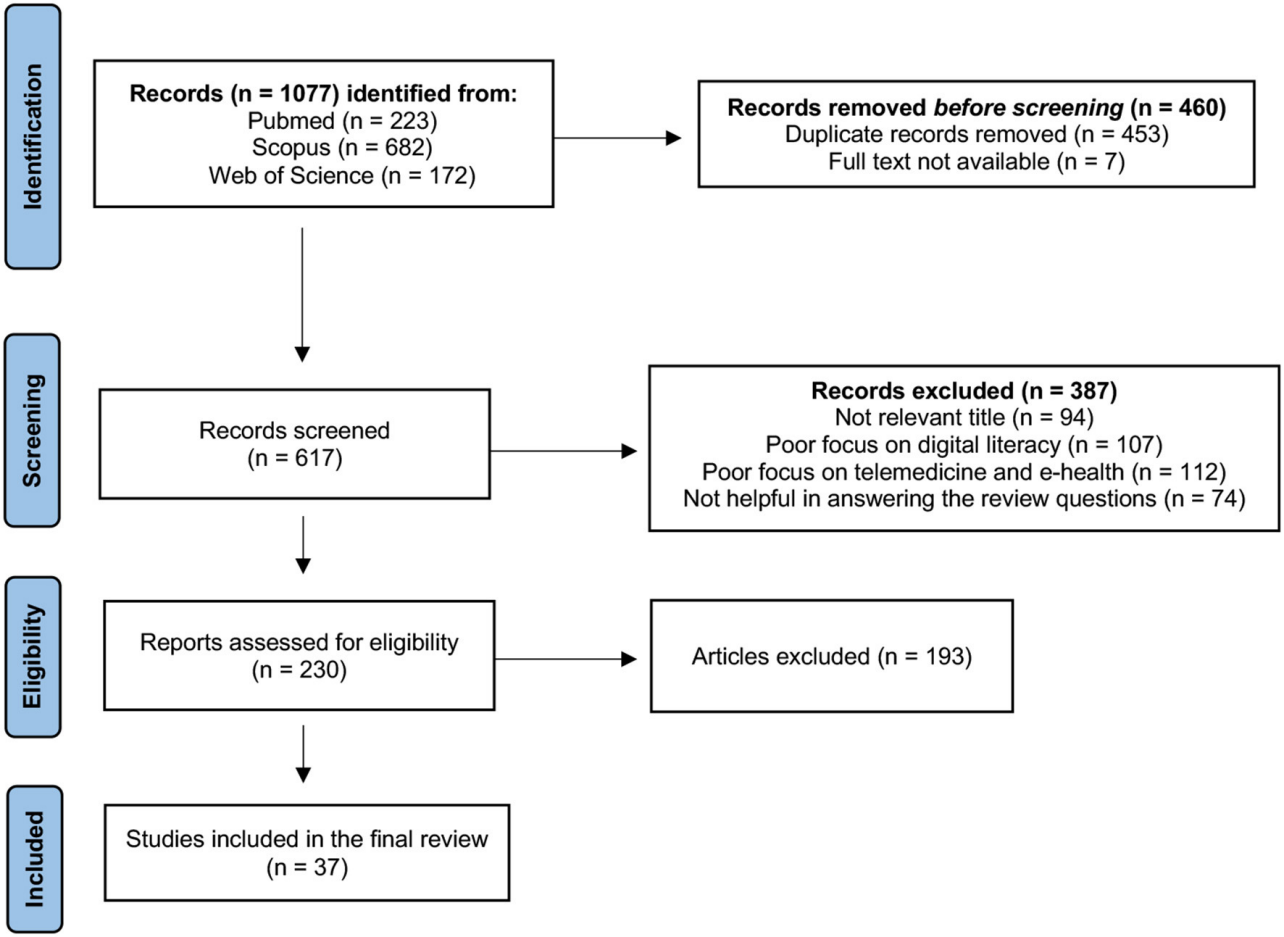
Article selection process.

Figure 1 shows the article selection process.

### 3.3. Quality evaluation

SANRA (Scale for the Assessment of Narrative Review Articles) (13) was employed for a quality check of the selected studies. SANRA includes 6 items: justification of the article's importance for the readership, statement of concrete/specific aims or formulation of questions, description of the literature search, referencing, scientific reasoning, and appropriate presentation of the data. A score from 0 to 2 is given for each item. The overall quality was determined as poor (score 0–6), moderate (7, 8, 10), or excellent (11–13). A total of 24 papers were found to be of moderate quality and 13 of excellent quality.

### 3.4. Summary of article pool

The search identified 37 articles suitable for inclusion in this review. The 37 papers were published between 2013 and 2022. Specifically, 1 article was published in 2013, 1 in 2016, 4 in 2018, 3 in 2019, 1 in 2020, 11 in 2021, and 16 in 2022. A total of 14 papers are literature reviews or papers in which the authors express their views on the topic (classic literature reviews, scoping reviews, systematic reviews, perspective papers), and 23 are papers in which actual research was conducted (mainly retrospective and survey studies). Given the large number of articles included in the review and the objective difficulty in discursively describing the results of all 37 papers, we briefly illustrated the main characteristics of the selected papers within Table 1. The articles were listed in order of year of publication.

**Table 1:** Summary of the content of the 37 articles included in the review.

**Authors, reference, and year of publication**	**Socio-environmental context**	**Type of article**	**Title**	**Purpose of the article**	**SANRA score**	**Conclusions of the article**
Choi and DiNitto (14)	USA	Original research	The digital divide among low-income homebound older adults: Internet use patterns, eHealth literacy, and attitudes toward computer/Internet use	Comparing the eHealth literacy and attitudes toward computer/Internet use of low-income homebound people aged 60 and older with that of their younger counterparts	8	Homebound low-income adults and the elderly have reduced opportunities to access e-Health services either due to limited digital literacy or economic or health circumstances
Jacobs et al. (15)	Worldwide	Systematic review	A systematic review of eHealth interventions to improve health literacy	Examining strategies designed to improve the health literacy of consumers of e-Health services	8	e-Health interventions specifically designed to improve health literacy can be delivered for people with different health conditions and risk factors. E-Health interventions are sometimes most effective in individuals with a very low level of digital literacy
Levin-Zamir and Bertschi (16)	Worldwide	Perspective article	Media Health Literacy, eHealth Literacy, and the Role of the Social Environment in Context	Clarifying the scope of the concepts of Media Health Literacy and e-Health Literacy	7	It is crucial to try as much as possible to ensure that everyone has access to media and digital tools, so that a virtuous alliance between healthcare services and technology can take place
Banbury et al. (17)	Canada, United States, Sweden, Norway, Australia, and Germany	Systematic review	Telehealth Interventions Delivering Home-based Support Group Videoconferencing: Systematic Review	Defining the feasibility and effectiveness of group videoconferences conducted by health professionals	11	The acceptability of group video conferencing for medical purposes tends to be high in all age groups, and poor digital literacy does not appear to be a barrier to the implementation of this health service delivery mode
van Houwelingen et al. (18)	Netherlands	Cross-sectional survey study + observational study	Understanding Older People's Readiness for Receiving Telehealth: Mixed-Method Study	Defining the level of digital health literacy of the elderly population and the factors that can predict its extent	11	The propensity of the elderly to use telemedicine services is directly predicted by their performance expectancy, effort expectancy, and perceived privacy or security. Self-efficacy and digital literacy play a major role in seniors' ability to use digital technology
MacLure and Stewart (19)	UK	Case study	A qualitative case study of ehealth and digital literacy experiences of pharmacy staff	Surveying the level of digital literacy of pharmacy staff	7	Promoting digital literacy is also of paramount importance for staff working in pharmacies, considering that a virtuous integration of hospital and pharmacy services is the basis of an efficient health service delivery system
Alam et al. (20)	Australia	Cross-sectional study	Determinants of access to eHealth services in regional Australia	Empirically investigating the current state and predictors of eHealth service access in regional Australia	8	Digital literacy is strongly correlated with the extent to which e-Health services are used and accepted
Azzopardi-Muscat and Sørensen (21)	Worldwide	Literature review	Toward an equitable digital public health era: promoting equity through a health literacy perspective	Defining the extent of the impact of digital technologies on health equity	7	It is essential to promote health literacy at all levels: individual, organizational, business, technical and political
Holt et al. (22)	Denmark	Cross-sectional study	Differences in the Level of Electronic Health Literacy Between Users and Nonusers of Digital Health Services: An Exploratory Survey of a Group of Medical Outpatients	Investigating how users and non-users of digital services differ with respect to skills in e-Health literacy	11	Age, gender, and education level are not factors related to greater or lesser propensity to use e-Health services, while digital and IT skills and health knowledge are
Lam et al. (23)	USA	Cross-sectional study	Assessing Telemedicine Unreadiness Among Older Adults in the United States During the COVID-19 Pandemic	Defining the prevalence of older adults not ready to access video or telephone telemedicine due to low digital literacy	7	Many elderly people are willing and even able to familiarize themselves with telemedicine, but for some categories of them (persons with dementia and socially isolated) the use of telemedicine seems objectively impracticable
Oh et al. (24)	United States, Germany, China, Italy, Sweden, Canada, Iran, and Bangladesh	Systematic review	Measurement of Digital Literacy Among Older Adults: Systematic Review	Assessing digital literacy among older adults	8	It is currently not easy to define precisely how digitally literate the elderly population is, requiring refinement of digital literacy assessment tools, which are currently still not fully adequate
Baker-Smith et al. (25)	USA	Single-center study	Impact of Social Determinants and Digital Literacy on Telehealth Acceptance for Pediatric Cardiology Care Delivery during the Early Phase of the COVID-19 Pandemic	Attempting to understand the degree of correlation between the acceptance of telemedicine by parents of children with heart disease and parents' digital literacy	10	Parents with experience in using video conferencing platforms are more willing to accept telemedicine services, so digital literacy may be a key factor in accessing telemedicine services
Abdulai et al. (26)	Africa (Ghana)	Cross-sectional survey	COVID-19 information-related digital literacy among online health consumers in a low-income country	Assessing the digital literacy of those who learned medical information about COVID-19 online	8	A good level of digital literacy is not sufficient to ensure beneficial use of the medical resources available online, as basic cultural qualities are also required to enable the user to identify valid and reliable scientific information
Boriani et al. (27)	Italy	Single-center study	Digital literacy as a potential barrier to implementation of cardiology tele-visits after COVID-19 pandemic: the INFO-COVID survey	Evaluating digital literacy among cardiology outpatients	10	Digital literacy levels are critical in determining the extent to which telemedicine services are used, and in the Cardiology field the level of confidence in technological means still tends to be low
Chung et al. (28)	USA	Cross-sectional study	The Role of Social Support in Telehealth Utilization Among Older Adults in the United States During the COVID-19 Pandemic	Investigating the role of social support for the use of telemedicine services among the elderly during the COVID-19 pandemic	9	One of the key tools to ensure full access to telehealth services is digital literacy support, which must also be pursued through social support
El Benny et al. (29)	Worldwide	Scoping review	Application of the eHealth Literacy Model in Digital Health Interventions: Scoping Review	Exploring how digital health interventions assess and evaluate the e-Health literacy model	8	Future Digital Health Interventions (DHIs) should assess the e-Health literacy model while developing or evaluating interventions
Samuels-Kalow et al. (30)	Worldwide	Perspective article	Digital disparities: designing telemedicine systems with a health equity aim	Exploring the mechanisms through which telemedicine can amplify or reduce disparities in health care	7	To effectively implement health literacy, structural, social and environmental barriers to understanding and using health information must be addressed
McAlearney et al. (31)	USA	Randomized controlled trial	Examining Patients' Capacity to Use Patient Portals: insights for Telehealth	Defining the factors determining the degree to which patients are able to use a portal intended for patients	12	It is essential that patients be provided with targeted training in the use of telehealth tools
Alkureishi et al. (32)	USA	Single-center study	Digitally Disconnected: Qualitative Study of Patient Perspectives on the Digital Divide and Potential Solutions	Understanding the causes and impact of the digital divide, who is responsible for it, and potential solutions	11	It is crucial that we invest in the implementation of digital literacy as a social determinant of health (SDOH), but on the other hand, given that a good portion of individuals will never cross the digital divide, it is also necessary to ensure low-tech methods of health care delivery
Anaya et al. (33)	Worldwide	Perspective article	Meeting them where they are on the web: addressing structural barriers for Latinos in telehealth care	Providing guidance on how to make telehealth equitable and accessible to all	8	Ensuring that the most vulnerable populations acquire digital skills would allow an important break down of barriers to the universality of telemedicine
Hsiao et al. (34)	USA	Cross-sectional retrospective study	Disparities in Telemedicine Access: A Cross-Sectional Study of a Newly Established Infrastructure during the COVID-19 Pandemic	Understanding patterns of telemedicine use after widespread deployment in order to identify potential disparities amplified by extensive and widespread use of telemedicine	9	It is critical to conduct adequate community outreach and education to ensure optimal digital literacy and subsequent equitable access to telemedicine
Cantor et al. (35)	Worldwide	Literature review	Effectiveness of Telehealth for Women's Preventive Services	Evaluating the effectiveness of telehealth services for women's preventive services for reproductive healthcare and interpersonal violence	7	Along with limited access to the Internet, low digital literacy is the key barrier to accessing telemedicine services
Maramba et al. (36)	Worldwide	Scoping review	The Role of Health Kiosks: Scoping Review	Trying to understand what the barriers and facilitators to the spread of health kiosks are	9	Given the ease of use of health kiosks, they represent an excellent way to deliver telemedicine services even to the low digitally literate
Berry et al. (37)	USA	Cross-sectional study	Patients' Perspectives on the Shift to Telemedicine in Primary and Behavioral Health Care during the COVID-19 Pandemic	Examining patients' perspectives on the use of telemedicine during the COVID-19 pandemic	8	Addressing the challenges of digital literacy is paramount to ensuring equitable access to telemedicine services
De Main et al. (38)	USA	Research article	Assessing the Effects of eHealth Tutorials on Older Adults' eHealth Literacy	Comparing the effectiveness of a multimedia tutorial versus a paper tutorial in improving the digital literacy of older adults	7	Digital literacy of the elderly is significantly more effective when practiced with multimedia tools
Mueller et al. (39)	USA	Retrospective cohort study	Disparities in telehealth utilization in patients with pain during COVID-19	Defining the sociodemographic characteristics of pain patients receiving telemedicine services during the COVID-19 pandemic	10	There is significant heterogeneity in access to telemedicine services, to some extent attributable to levels of digital literacy
Mather et al. (40)	Australia	Cross-sectional survey	eHealth Literacy of Australian Undergraduate Health Profession Students: A Descriptive Study	Exploring the digital literacy of undergraduate health professions students	10	Health professions curricula should contain specific teachings on digital literacy to create professionals ready to work in a future where telemedicine will permeate health care
Nelson et al. (41)	USA	Original research	A 3-Item Measure of Digital Health Care Literacy: Development and Validation Study	Developing and validating a scale capable of assessing digital health care literacy	10	A screening tool such as a scale that can measure digital literacy in health care would be a key resource for identifying patients needing support to enjoy the benefits of telemedicine
Livingood et al. (42)	US	Original research	Comparative study of different SES neighborhood clinics for health literacy and internet access	Investigating the relationship between the levels of digital literacy and efforts made by university affiliated primary care clinics to implement the quality of telemedicine services	11	Health and public health clinics need to be aware of the difference in health literacy and Internet access when implementing technology-based services, keeping in mind that even patients of high socioeconomic status may be poorly digitally literate
Gillie et al. (43)	Worldwide	Literature review	Telehealth Literacy as a Social Determinant of Health: A Novel Screening Tool to Support Vulnerable Patient Equity	Proposing a telehealth literacy screening tool for the elderly	7	Telehealth literacy must be formally recognized as an important social determinant of health (SDOH)
Gallegos-Rejas et al. (44)	Worldwide	Perspective article	A multi-stakeholder approach is needed to reduce the digital divide and encourage equitable access to telehealth	Proposing practical solutions to reduce the digital divide and encourage equitable access to telehealth	7	Digital divide reduction strategies must be multi-stakeholder
Lee et al. (45)	USA	Single-center study	Understanding and Addressing the Digital Health Literacy Needs of Low-Income Limited English Proficient Asian American Patients	Attempting to define how language difficulties impact access to eHealth services	11	The barrier to accessing telemedicine services secondary to limited digital literacy is likely to be amplified by the language difficulties of the population who do not speak the language of the country in which they reside
Rasekaba et al. (46)	India	Mixed-method cross-sectional focus group and survey-based study	Exploring Telehealth Readiness in a Resource Limited Setting: Digital and Health Literacy among Older People in Rural India (DAHLIA)	Examining digital and health literacy in a sample of 150 older adults residing in two rural areas of India	8	Digital literacy levels are lower in rural areas. One possible solution to this problem is the implementation of social support from family and health care institutions and the dissemination of user-friendly technology
AlKhanbashi and Zedan (47)	Saudi Arabia	Observational cross-sectional study	Telemedicine Policy Availability and Awareness: Directions for Improvement	Assessing the level of digital literacy of health workers working in ambulatory care clinics	8	It is critical that inequalities in digital literacy be given the utmost consideration when designing and implementing telemedicine programs
Ng et al. (48)	USA	Original research	Accessibility and utilization of telehealth services among older adults during COVID-19 pandemic in the United States	Investigating factors associated with accessibility and use of telemedicine among older adults during the COVID-19 pandemic	11	Digital literacy, along with socioeconomic status, is an important indicator of telehealth accessibility in the elderly
Lopez de Coca et al. (49)	Worldwide	Systematic review	Bridging the Generational Digital Divide in the Healthcare Environment	Assessing the extent of the digital divide of patients in relation to the health care environment	8	Levels of e-Health literacy still need to be increased, especially among the elderly population, in order to prevent poorly digitally literate individuals from being misled by the health information they learn through technological means
van Kessel et al. (50)	Worldwide	Scoping review	Digital Health Paradox: International Policy Perspectives to Address Increased Health Inequalities for People Living With Disabilities	Exploring the potential benefits of digital technologies for the global population, with special reference to people with disabilities, using the autism community as a case study	8	Although we see futuristic scenarios in relation to the use of digital technologies, it is vital today that we implement digital health literacy policies that can make people keep up with the rapid advances in technology

## 4. Discussion

The insights provided by the analysis of the reviewed articles are numerous. A first fundamental point seems to be the importance that is universally recognized to digital literacy as the key to ensuring an equitable distribution of health services in the society of the third millennium, permeated by digitization and technologization (16, 20, 27–29, 33, 35, 37, 47, 50). In order for it to succeed, it is essential that the promotion of digital literacy should not be a temporary and circumscribed measure, but on the contrary should take on a structural character and be explored at all levels of the organization of society (21). This is important, for example, when considering the fact that those who due to economic or logistical difficulties (homebound) have limited access to technological resources (14). A digital literacy project aimed at these segments of the population cannot be fruitful unless there is a concomitant effort to support them economically and socially. Also consider the linguistic aspect (45). It is perfectly useless to digitally literate an individual who then, due to lack of understanding of the language, is unable to use the electronic tools he or she has become capable of using.

Intimately associated with this concept is the principle that ensuring equity of access to digital health through the enhancement and development of digital literacy must be a priority felt by multiple stakeholders: consumers (patient and carers), consumer advocacy groups, health service staff (clinicians, nurses, pharmacists), health services (providers), policy-makers/funders, researchers, and industries (44). This is because telemedicine and medical care provided through digital tools in general is now officially and definitively a concrete and tangible reality, so much so that it is universally recognized as a social determinant of health (SDOH) (30, 43). A very interesting idea would be to provide for the introduction of specific teachings on digital literacy within school curricula.

It is very important to note, however, that like any ambitious project, that of adapting digital literacy to the levels of technology that characterize our society today must come to terms with realism. This means that providing for an extension and capillarization of digital skills would risk further exacerbating technological evolution, thus risking leading to a widening of the “digital divide” secondary to the gap that could arise from the speed of growth of digital skills (fast) and the speed of growth of technology (very fast). In other words, there would be a risk of favoring technology that evolves too fast compared to how fast digital skills evolve (23).

It is therefore essential to pursue the goal of supporting increasing digital literacy in a prudent and reasoned manner, also in view of the fact that e-Health services are often, paradoxically, particularly effective in low digitally literate individuals (15). This paradoxical effect is confirmed by the fact that health kiosks represent the perfect paradigm of the digital medicine tool suitable for the person with low digital literacy (36). The reason is simple: although they are highly technological and complex tools, they have a highly intuitive interface for use of which no special computer skills are required. Therefore, some might argue that it would be wrong to concentrate forces in implementing digital literacy of the population, a strategy that would exclude categories of people who are objectively difficult to literate, and that it would be wiser, on the contrary, to try to simplify e-Health technological tools as much as possible, so that their use can be made within the reach of everyone, even those who are not familiar with technological means. Arguably, increasing the digital skills of the general population and developing e-Health technologies with the highest benefit-to-complexity ratio are operational strategies that must be pursued together. In any case, it should be considered how it is objectively inadvisable to take the simplification of e-Health benefits to extreme limits, since simplifying a technological measure often also means preventing it from unfolding its full potential.

Another interesting food for thought that emerged from the review is how to identify individuals who may benefit from digital literacy programs. Despite the fact that very often the category of people unable to independently use digital tools in the service of health coincides with the elderly population, this correspondence is not always true, depending, as the review clearly demonstrated, on numerous other factors. Even the parameter of socioeconomic status is not accurate in defining who is reliably digitally competent and who is not. In fact, not always those with high incomes (and therefore likely to have access to high-level technological tools) possess a sufficient degree of digital literacy to make beneficial use of e-Health services (42). It is therefore essential to develop screening tools that can accurately identify the population groups in need of digital literacy interventions (24, 41). These tools already exist, but, as the literature consulted shows, they need to be considerably implemented. As for developing countries, it should be pointed out that they rely heavily on ICT (Information Communication Technology) tools for their economic survival, competition and progress, which is why it is even more important for digital literacy to be implemented in these areas (51).

It is also interesting to note that, based on the review conducted, it can be concluded that underlying the need to ensure uniform digital literacy should not only be the desire to ensure “equitable” healthcare, but also to prevent the development of “unfair” healthcare. Indeed, those who are poorly digitally literate not only have fewer options in terms of access to care, but are also more vulnerable to the negative and detrimental effects that can result from finding unreliable online information or the product of fraudulent scientific research (49). From this we can see that digital literacy programs are much more than teaching how to become familiar with the technological tools. In fact, making people digitally literate is primarily about teaching them to become aware of the scope and limitations of technological tools in the service of digital health. Clearly, the likelihood of success of this purpose depends largely on the cultural background and knowledge base of individuals (26), but it should not be assumed that individuals with high levels of education are always able to discern what is scientifically valid and what is not, especially if the information is learned through a technological tool with which they are not familiar.

In summary, then, we can conclude how digital literacy is an essential element in the development of equitable digital medicine. However, this is a complex road ahead, given the complexity of the underlying socioeconomic and cultural scenarios, the critical issues in identifying the target population, and the need for a multidisciplinary and multiple stakeholder approach.

## 5. Conclusion

As a final consideration, we can note how it is not only important to implement digital education programs that can bridge as much of the “digital divide” as possible, but how it is equally important to plan for evaluation studies of the effectiveness of such programs in the immediate future.

## Data availability statement

The original contributions presented in the study are included in the article/supplementary material, further inquiries can be directed to the corresponding author.

## Author contributions

LC: conceptualization and writing original draft. FG, PB, and GN: writing, reviewing, and editing. AS and GR: supervision and coordination. All authors contributed to the article and approved the submitted version.
